# Research and Development of Delay-Sensitive Routing Tensor Model in IoT Core Networks

**DOI:** 10.3390/s21113934

**Published:** 2021-06-07

**Authors:** Oleksandr Lemeshko, Jozef Papan, Oleksandra Yeremenko, Maryna Yevdokymenko, Pavel Segec

**Affiliations:** 1 V.V. Popovskyy Department of Infocommunication Engineering, Kharkiv National University of Radio Electronics, 61166 Kharkiv, Ukraine; oleksandr.lemeshko.ua@ieee.org (O.L.); oleksandra.yeremenko.ua@ieee.org (O.Y.); maryna.yevdokymenko@ieee.org (M.Y.); 2 Department of InfoCom Networks, University of Žilina, 010 26 Žilina, Slovakia; pavel.segec@fri.uniza.sk

**Keywords:** Internet of Things (IoT), core network, delay-sensitive routing, Quality of Service (QoS), average end-to-end delay

## Abstract

In the article, we present the research and development of an improved delay-sensitive routing tensor model for the core of the IoT network. The flow-based tensor model is considered within the coordinate system of interpolar paths and internal node pairs. The advantage of the presented model is the application for IoT architectures to ensure the Quality of Service under the parameters of bandwidth, average end-to-end delay, and the probability of packet loss. Hence, the technical task of delay-sensitive routing is formulated as the optimization problem together with constraints and conditions imposed on the corresponding routing variables. The system of optimality criteria is chosen for an investigation. Each selected criterion concerning the specifics of the demanded routing problem solution aims at the optimal use of available network resources and the improvement of QoS indicators, namely, average end-to-end delay. The analysis of the obtained routing solutions under different criteria is performed. Numerical research of the improved delay-sensitive routing tensor model allowed us to discover its features and proved the adequacy of the results for the multipath order of routing.

## 1. Introduction

The constant increase in requirements for communication networks and, above all, the maintenance of Quality of Service (QoS) dictates the need for continuous improvement in existing technologies and protocols. It leads to their evolutionary revision and additional theoretical foundations. By definition, the theory of telecommunications is based on system approach principles when a network as an object of research, analysis, and synthesis is considered as a complex organizational and technical system. The main factors of network complexity include the following [1,2,3]:

- Scale, which refers to both the number of network elements (sensors, terminals, switches, routers, servers, links, etc.) and the set of functions that the network can support;
- Territorial distribution, due to the location of interacting network elements at a considerable distance;
- High dynamics, as the state of the network, for example, its topology and utilization, can change in real time;
- Heterogeneity, which is the use within one network of quite different principles and operation conditions of communication technologies, protocols, and switching equipment.

As the analysis showed, the role of a mathematical apparatus that can provide a holistic network description as a complex multidimensional system can claim tensor analysis of networks. Its basics were proposed by American research engineer G. Kron [4,5] primarily for circuits. Recently, such solutions have been supplemented by scientific and applied results to analyze and study various technical, economic, and social systems with complex architecture [6,7,8,9].

Due to the direct analogy of electrical and communication network processes, the tensor analysis apparatus applies to a wide range of traffic management and routing [10,11,12,13,14,15,16]. However, in terms of their structural and functional construction, communication networks are more complex systems than electrical circuits. Therefore, the methodology of Kron can be considered the first step on the adaptation path of tensor calculation and analysis ideas and principles for network research and development.

Differences in the tensor description of electrical circuits and communication networks, as a rule, occur at the initial stages of geometrization and metrization [17,18,19,20,21,22]. Consequently, in the electrical circuits tensor analysis process, G. Kron and his followers proposed using only two types of orthogonal coordinate systems—the basis of network edges and the basis of circuits and node pairs [17,18,19,20]. However, researchers in communications find an applied interpretation and utilization of other coordinate systems, which mathematicians introduced on one-dimensional networks (graphs). For example, this concerns the basis of circuits, cuts, interpolar paths, and internal node pairs [17,18,19,20,21,22]. The use of new coordinate systems in tensor modeling any system introduces an alternative aspect of the study object, opening additional opportunities and prospects for research and obtaining new beneficial results for science and practice.

Therefore, this article aims to study the tensor model of delay-sensitive routing in communication networks proposed in [23], which is presented in the coordinate system of interpolar paths and internal node pairs. The proposed tensor model contains new mathematical expressions for calculating end-to-end Quality of Service indicators, namely average end-to-end delay and packet loss probability, which are valid in conditions of implementation dynamic and multipath routing strategies. Furthermore, these expressions use optimality criteria or constraints imposed on routing variables when increasing or ensuring a given QoS level during traffic management and routing optimization.

The remainder of this paper is structured as follows: Section 2 contains a summary of the latest knowledge in QoS routing in IoT and sensory networks and provides an analysis of existing solutions. In addition, Section 2 discusses their peculiarities. Section 3 proposes the flow-based routing model in a core communication network, taking into account probable packet losses. Section 4 focuses on tensor network description and the formalization of Quality of Service assurance conditions. Section 5 provides the analysis of the optimality criteria system for QoS routing problem solving. Section 6 contains the evaluation of the presented tensor flow-based routing model and comparative analysis of QoS routing problem solutions under different optimality criteria. Section 7 presents the conclusions of the work and directions for future research.

## 2. Related Research Analysis

Due to the IoT devices’ constant growth, the underlying telecommunication networks (IoT networks core) must support and provide Quality of Service [24,25]. Moreover, they must be adaptive to IoT traffic nature and characteristics, so telecommunication infrastructures must be designed by the traffic specifics they transmit. The different data types, such as voice, video, dynamic IoT data, etc., have peculiarities and corresponding QoS requirements for transmission via the IoT infrastructure. Therefore, it should be mentioned that IoT traffic significantly differs, and its QoS demands must be considered when designing the IoT core network.

In the process of QoS routing in modern multiservice networks, including IoT and Wireless Sensor Networks (WSNs), it is crucial to ensure the Quality of Service on only one indicator of Network Performance (NP). However, when servicing packets of most applications, it is necessary to provide QoS on many indicators related to bandwidth, time, and reliability [26,27]. For example, packet flows of multimedia applications are sensitive both to the allocated bandwidth and to the level of packet delays, etc. Considering this when solving QoS routing problems, it is necessary to provide the value of not one but many indicators of Quality of Service.

Based on the analysis of existing solutions for QoS routing in IoT and WSN [28,29,30,31,32,33,34,35], the classification of the main types of solutions to ensure the Quality of Service level in telecommunication networks that support and maintain them has been performed (Table 1).

First of all, solutions regarding QoS routing in core networks were generally considered [28,29,30]. Today, SDN-based solutions prevail among the solutions, which make it possible to effectively implement sophisticated network management and QoS routing mechanisms on the control plane, for example, using blockchain technologies or multicriteria optimization [29,30]. In addition, these solutions, as a rule, are complex and allow guaranteeing QoS in several indicators, primarily in terms of end-to-end packet delay and bandwidth.

Additionally, all considered solutions can be divided into two broad categories: heuristic and optimization [28,29,30,31,32,33,34,35]. The most promising is precisely the optimization approach, which, when solving QoS routing problems, is aimed at optimal network resources.

However, the QoS routing solutions in IoT and WSN revealed that delay requirements must always be fulfilled [31,32,33,34,35]. At the same time, the solutions based on the softwarized network management and QoS routing in IoT and WSN aimed at considering all the needed QoS indicators, namely, delay, loss, and bandwidth.

A common feature of the vast majority of promising theoretical solutions to QoS routing problems is their formulation in optimization form [16,17,18,19,20,21,22,31,33,35,36,37,38,39,40], which helps increase the efficiency of using a valuable network resource for the practical implementation of these solutions. Depending on the aspect and detail of the consideration of the routing task and the completeness of external and internal factors concerning the network, optimization problems can belong to a variety of classes and types.

Quite often, routing problems in the network with different settings correspond to the optimization problems of mathematical programming [16,17,18,19,20,21,22,36], while the simplest of them are usually formulated as problems of linear programming [36,37]. For example, these solutions include optimization models of multipath routing with load balancing, which indirectly affects the improvement of network Quality of Service. However, when it comes to meeting the requirements for quantitative values of fundamental QoS indicators in solving routing problems, there is a need to introduce into the model or method of routing in one or another form the mathematical expressions to calculate these QoS indicators. Given that the formulas for calculating QoS indicators are exclusively nonlinear functions of network parameters and traffic characteristics, the optimization routing problems themselves acquire a nonlinear form, which requires the use of appropriate methods for their solution, such as nonlinear programming [16,17,18,19,20,21,22,23]. Furthermore, if it is necessary to consider the network dynamics in the formalization and solution of optimization routing problems, most network parameters also become a function of time [38]. In this case, the optimal control methods are actively used in the calculation process.

Hence, the analysis performed allows us to conclude that on the one hand, the mechanisms underlying effective technological solutions should be comprehensive and fulfill the requirements on the entire set of necessary QoS indicators, taking into account the specifics of IoT traffic. On the other hand, using the SDN-based approach will make it possible to implement the proposed sophisticated models and methods.

According to problem areas of QoS routing in IoT and WSNs core networks technical tasks, the following demands to promising solutions can be formulated:

- Taking into account the multiflow and multimedia nature of modern network traffic, depending on the type of which it is necessary to ensure the Quality of Service for many indicators of network performance (delay, bandwidth, packet loss, jitter);
- Providing a complex solution to ensure a given level of QoS on the set of NP indicators;
- Ensuring the optimality and scalability of routing solutions with QoS support.

Full and comprehensive satisfaction of the listed set of technical requirements can be provided only by improving existing and developing new mathematical models and methods of QoS routing, which would also form the basis of the mathematical, algorithmic, and software background of promising routers, servers, switches, and SDN controllers aimed at using in the IoT core network.

As stated above, the research goal is a study of the tensor model of delay-sensitive routing in the core network presented in the coordinate system of interpolar paths and internal node pairs. Therefore, the objectives of the work are as follows:

- Formulation of the flow-based routing model in a communication network taking into account packet losses;
- Performing the tensor description of the network and formalization of Quality of Service assurance conditions;
- Selection and analysis of optimality criteria system of QoS routing problem solving;
- Evaluation of the tensor flow-based routing model and comparative analysis of QoS routing problem solutions.

## 3. Flow-Based Routing Model in a Communication Network Taking into Account Probable Losses of Packets

The solution described in works [17,20] and based on the description of the network structure by an oriented graph Γ=(U,W) was chosen as the basis of the flow-based model of routing in the network. The set of vertices $U=\left\{u_{i},i=\bar{1,m}\right\}$ simulates network routers, and the set of graph edges $W=\left\{w_{i,j},\;i,j=\bar{1,m};\;i\neq j\right\}$ describes communication links. Denote by $\varphi_{i,j}$ the capacity of the link in packets per second (1/s), which is determined by the bandwidth of the jth network interface on the ith router. Accordingly, the numbering of links is double and is set through the number of adjacent routers.

In general, a set of packet flows K circulates concurrently in a network, for the routing of which a set of routing variables $x_{i,j}^{k}$ must be calculated. Each control variable determines the fraction (part, share) of the kth packet flow, which is sent from the ith to the jth router through the appropriate interface. Depending on the type of routing implemented in a network, the following conditions are imposed on routing variables:

- When implementing single path routing

(1) $$x_{i,j}^{k}\in \left\{0,1\right\},$$

- When implementing multipath routing

(2) $$0\leq x_{i,j}^{k}\leq 1.$$

Denote by $p_{i,j}^{k}$ the packet loss probability of the kth flow is caused by the queue buffer overload on the jth interface of the ith router. Then, the intensity of the kth flow of packets that are rejected (lost) on the jth interface of the ith router can be calculated using the following formula:

(3) $$r_{i,j}^{k}=\lambda_{k}^{req}x_{i,j}^{k}p_{i,j}^{k},$$

where $\lambda_{k}^{req}$ is the average intensity of the kth packet flow, which directly determines the requirements for the Quality of Service level in terms of bandwidth.

Then, the intensity of the transmitted lossless packets of the kth flow in the link, which is modeled by the edge $w_{i,j}$, is determined as follows:

(4) $$\lambda_{i,j}^{k}=\lambda_{k}^{req}x_{i,j}^{k}\left(1-p_{i,j}^{k}\right).$$

Furthermore, the flow conservation conditions considering the probable loss of packets on each of the network routers take the following form [17,23]:

(5) $$\left\{ \begin{array}{c} |\sum_{j:w_{i,j}\in W}x_{i,j}^{k}=1,\;k\in K,\;u_{i}=s_{k};\\ |\sum_{j:w_{i,j}\in W}x_{i,j}^{k}-\sum_{j:w_{j,i}\in W}x_{j,i}^{k}\left(1-p_{j,i}^{k}\right)=0,\;k\in K,\;u_{i}\neq s_{k},d_{k};\\ |\sum_{j:w_{i,j}\in W}x_{j,i}^{k}\left(1-p_{j,i}^{k}\right)=b^{k},\;k\in K,\;u_{i}=d_{k}, \end{array}\right.$$

where $s_{k}$ is the source router (sender) of packets; $d_{k}$ is the destination router (receiver) of packets of the kth flow; and $b^{k}$ is the fraction of the kth flow of packets that have been successfully transmitted in the network from the source router to the destination.

The type of mathematical expression used to calculate the packet loss probability is generally influenced by the flow characteristics (intensity, average packet length, etc.) and interface parameters (bandwidth, queue buffer size, selected packet service discipline). For example, in the case of modeling the operation of the jth interface of the ith router queuing system of the type M/M/1/N with failures, the probability of packet loss of the kth flow can be calculated as follows:

(6) $$p_{i,j}^{k}=\frac{\left(1-\rho_{i,j}\right){\left(\rho_{i,j}\right)}^{N}}{1-{\left(\rho_{i,j}\right)}^{N+1}},$$

where the utilization factor of the jth interface on the ith router ($\rho_{i,j}$) is determined by the formula

(7) $$\rho_{i,j}=\frac{\sum_{k\in K}\lambda_{k}^{\left\langle req\right\rangle }x_{i,j}^{k}}{\varphi_{i,j}}.$$

To prevent links overload of a communication network in terms of their bandwidth, the variables $x_{i,j}^{k}$ are subject to the following restrictions:

(8) $$\sum_{k\in K}\lambda_{k}^{\left\langle req\right\rangle }x_{i,j}^{k}<\varphi_{i,j}.$$

## 4. Tensor Description of the Network and Formalization of Quality of Service Assurance Conditions

The leading indicators of network Quality of Service include bandwidth, average delay, and packet loss probability [26,27]. In the general case, the conditions for providing QoS on these indicators are as follows:

(9) $$\lambda_{k}^{\left\langle req\right\rangle }<\varphi_{k},\;\tau_{MP}^{k}\leq \tau_{\left\langle TH\right\rangle }^{k}\;\mathrm{and}\;p_{\left\langle TH\right\rangle }^{k}\geq p^{k},$$

where $\varphi_{k}$ is the network bandwidth allocated by the kth packet flow;

$\tau_{MP}^{k}$ is the average end-to-end delay of the kth packet flow in the network;

$p^{k}$ is the packet loss probability of the kth flow;

$\tau_{\left\langle TH\right\rangle }^{k}$ and $p_{\left\langle TH\right\rangle }^{k}$ are the permissible (threshold) values for the average end-to-end delay and the packet loss probability of the kth flow in the network.

Conditions to prevent overloading of links (8) is one of the variants of requirements for providing QoS in terms of network bandwidth. In addition, according to models (1)–(8) [23]:

(10) $$p^{k}=1-b^{k}.$$

The average end-to-end delay of packets of any flow transmitted between a given pair of routers using a set of routes P is calculated by the following formula:

$$\tau_{MP}=\sum_{p=1}^{\left|P\right|}x_{p}\tau_{p},$$

where $x_{p}$ is the fraction of the packet flow that was successfully delivered to the receiving router via the pth path;

$\tau_{p}$ is the average delay of packets transmitted over the pth path in the network;

|P| is the power (size) of the set P, the value of which determines the total number of paths available for routing.

In the general case, the expression can be used for the calculation:

$$x_{p}=\frac{\lambda_{p}}{\lambda^{\left\langle req\right\rangle }b},$$

where $\lambda_{p}$ is the intensity of the flow of packets that were successfully delivered to the receiving router via the pth path.

The mathematical expression for the calculation $\tau_{MP}^{k}$ can be obtained in the process of tensor generalization of the model (1)–(8) [23]. The network links are additionally represented by a set of edges $V=\left\{v_{z};z=\bar{1,n}\right\}$, where n is the total number of links in the network. In addition to double link numbering, the continuous (single) numbering of links is introduced for the further tensor representation of the network.

Network nodes (routers) through which the kth packet flow incomes or outgoes, the network will be called poles. The following parameters can characterize a connected network:

κ(S) is the number of basis interpolar paths in the network S;
ϑ(S) is the number of basis internal node pairs.

At the same time, the set of internal node pairs includes all node pairs except for the pole one. Then, the topological invariants of a connected one-dimensional network S are interconnected by the following dependencies:

(11) κ(S)=n−m+2; ϑ(S)=m−2, n=κ(S)+ϑ(S).

As shown in [10,11,12,13,14,15,16,17,18,19,20,21,22], the network structure defines a discrete *n*-dimensional geometric space. In the introduced space for each individually selected packet flow, for which the conditions of QoS assurance will be obtained, the network will be described by a mixed bivalent tensor [19,20,21]:

(12) Q=T⊗Λ,

where ⊗ is the tensor multiplication operator;

T is the univalent covariant tensor of average packet delays;

Λ is the univalent contravariant tensor of flows’ average intensities in the coordinate paths of the network.

The coordinates of these tensors (12) are the average delay $\tau_{j}$ of the packets of the kth flow over the jth coordinate path (measured in seconds, s); the average intensity $\lambda^{i}$ of the kth flow of packets transmitted over the ith coordinate path (1/s). The index “k” in tensor expressions for clarity will be omitted.

The components of the mixed bivalent tensor (12) are connected by metric tensors [23]:

(13) T=EΛ and Λ=GT,

where E is the twice covariant metric tensor, while G is the twice contravariant metric tensor.

In what follows, two types of coordinate systems (CS) will be used to represent the tensors introduced in expressions (12) and (13):

- The basis of edges $\left\{v_{z},z=\bar{1,n}\right\}$, which are projections of tensors that will be denoted by an index v;
- The basis of interpolar paths $\left\{\gamma_{i},i=\bar{1,\kappa }\right\}$ and internal node pairs $\left\{\varepsilon_{j},j=\bar{1,\vartheta }\right\}$, in which the projections of the tensor will be denoted by the index γε.

In [23], it is shown that within the tensor description of the network, it is possible to obtain an expression for calculating the average end-to-end delay of packets of the kth flow, which is included in the set of QoS conditions (9):

(14) $$\tau_{MP}^{k}=\frac{1}{\lambda_{k}^{\left\langle req\right\rangle }b^{k}}\left(\Lambda_{\gamma }^{t}E_{\gamma \varepsilon }^{\left\langle 1\right\rangle }\Lambda_{\gamma }+\Lambda_{\gamma }^{t}E_{\gamma \varepsilon }^{\left\langle 2\right\rangle }\Lambda_{\varepsilon }\right).$$

In Expression (14), ${\left[\cdot \right]}^{t}$ is the operation of the matrix transposition; 

$\Lambda_{\gamma }$ and $\Lambda_{\varepsilon }$ are the projections of the flow intensity tensor Λ (12) in the coordinate system of the interpolar paths and internal node pairs, respectively, which are represented by vectors of size κ×1 and ϑ×1;

$E_{\gamma \varepsilon }^{\left\langle 1\right\rangle }$ and $E_{\gamma \varepsilon }^{\left\langle 2\right\rangle }$ are matrices of size κ×κ and κ×ϑ, which are blocks of the metric tensor projection in the coordinate system of interpolar paths and internal node pairs:

(15) $$E_{\gamma \varepsilon }=\|\begin{array}{ccc} E_{\gamma \varepsilon }^{\left\langle 1\right\rangle } & | & E_{\gamma \varepsilon }^{\left\langle 2\right\rangle }\\ --- & + & ---\\ E_{\gamma \varepsilon }^{\left\langle 3\right\rangle } & | & E_{\gamma \varepsilon }^{\left\langle 4\right\rangle } \end{array}\|.$$

Similarly, Projection (15) is obtained by the rule of covariant transformation:

(16) $$E_{\gamma \varepsilon }=C^{t}E_{v}C,$$

where C is the n×n matrix that determines the law of contravariant coordinate transformation when changing the introduced coordinate systems;

$E_{v}$ is the diagonal the n×n matrix, the coordinates of which are directly determined by the models of flows and service disciplines at the interfaces of network routers.

For example, when the operation of network router interfaces is simulated by the M/M/1/N queuing system, the average packet delay in the ith network link, which is the corresponding tensor T projection in the coordinate system of edges ($T_{v}$), can be calculated using the following formula [23]:

(17) $$\tau_{i}=\frac{\rho_{i}-\rho_{i}^{N_{i}+2}-\left(N_{i}+1\right)\rho_{i}^{N_{i}+1}\left(1-\rho_{i}\right)}{\lambda_{i}\left(1-\rho_{i}^{N_{i}+1}\right)\left(1-\rho_{i}\right)},$$

where $\varphi_{i}$ and $\rho_{i}=\frac{\lambda_{i}}{\varphi_{i}}$ are the bandwidth and utilization coefficient of the ith network link, respectively;

$\lambda_{i}$ is the total intensity of all packet flows sent to the ith network link; 

$N_{i}$ is the size of the queue buffer on the ith network interface.

Then, the matrix coordinates of the twice covariant metric tensor projection $E_{v}$ can be specified using the following expressions [17,23]:

(18) $$e_{ii}^{v}=\frac{\rho_{i}-\rho_{i}^{N_{i}+2}-\left(N_{i}+1\right)\rho_{i}^{N_{i}+1}\left(1-\rho_{i}\right)}{\lambda_{i}\left(1-\rho_{i}^{N_{i}+1}\right)\left(1-\rho_{i}\right)\lambda_{v}^{i}},$$

where $\lambda_{v}^{i}$ is the intensity of the kth packet flow in the ith network link; i.e., the flow, which is considered to form a tensor model (12).

The projections of the metric tensors E and G depend on the values of the routing variables as follows:

(19) $$\lambda_{z}=\sum_{k\in K}\lambda_{k}^{\left\langle req\right\rangle }x_{i,j}^{k}\;\mathrm{and}\;\lambda_{v}^{z}=\lambda_{k}^{\left\langle req\right\rangle }x_{i,j}^{k}\left(1-p_{i,j}^{k}\right).$$

Expression (19) determines the intensities of aggregated and separate kth flows in the same network link, which is modeled by an edge $v_{z}$ within the continuous numbering, and by an edge $w_{i,j}$ in the case of double numbering.

Therefore, following Requirement (9) and Expression (14), the condition of ensuring the Quality of Service on the average end-to-end packet delay in the network will take the form:

(20) $$\tau_{MP}^{k}\lambda_{k}^{\left\langle req\right\rangle }b^{k}\geq \Lambda_{\gamma }^{t}E_{\gamma \varepsilon }^{\left\langle 1\right\rangle }\Lambda_{\gamma }+\Lambda_{\gamma }^{t}E_{\gamma \varepsilon }^{\left\langle 2\right\rangle }\Lambda_{\varepsilon }.$$

Condition (20) connects the main QoS indicators provided by the network, as the improvement of one indicator may affect the value of another. QoS Conditions (8), (10), and (20) act as additional restrictions imposed on routing variables $x_{i,j}^{k}$.

## 5. Optimality Criteria System of QoS Routing Problem Solving

An essential point in the formulation, and later in solving the routing problem in the network, is the choice (formulation) of the criterion of optimal routing solutions. Traditionally, its content should have a clear physical interpretation in terms of the routing process. Moreover, its appearance and form should focus on obtaining the desired solution with minimal computing costs and in real time, i.e., fit into existing routing table update timers that amount to tens of seconds [26,27]. At present, there is no single form of the optimality criterion of routing solutions, which would satisfy most of its requirements. Given this, researchers, depending on the purpose of the study, use a robust system of criteria for optimal routing solutions [37], each of which has certain advantages and disadvantages that determine its direction of priority use in certain conditions of the multiservice communication network.

The optimality criteria of routing solutions, which will later find their use in this work, will be divided into three groups. The first group of criteria is related to the minimization of the network links utilization (7). This has a positive effect on the numerical values of the main QoS indicators (6) and (17). The second group of optimality criteria covers finding the extremum directly of specific QoS indicators if the applied mathematical model allows providing their calculation in analytical form. Finally, the optimality criteria of the third group are based on the combined consideration of both the network link utilization and the exact value of the QoS indicators.

An example of the optimality criterion of the first group is the minimum of the following linear objective function:

(21) $$J=\sum_{k\in K}\sum_{w_{i,j}\in W}h_{i,j}^{x}\lambda_{k}^{\left\langle req\right\rangle }x_{i,j}^{k},$$

where $h_{i,j}^{x}$ is the link routing metric which connects the ith and jth network routers.

Traditionally, routing metrics are related to the functional parameters of communication links. However, in the case where $h_{i,j}^{x}=1$, the solution of the routing problem for each kth packet flow is usually a path with a minimum number of hops. However, if the metric $h_{i,j}^{x}=\varphi^{*}/\varphi_{i,j}$ is used, the actual objective function (21) will determine the weighted sum of the utilization of all network links. In the case of small values of the weighting coefficient $\varphi^{*}$, the use of such a metric leads to the calculation of paths with the lowest total metric, i.e., with the highest bandwidth. In the case of increasing the values of $\varphi^{*}$ according to the minimization of the (21) results, the calculated paths will again be more critical to the number of hops. In existing protocols, such as OSPF or IGRP/EIGRP, the weighting coefficient $\varphi^{*}$ takes values $10^{8}$ or $10^{7}$. The work [39] considers quadratic or linear-quadratic variants of the optimality criterion (21), the application of which focuses on ensuring a more balanced use of the network link resource depending on the values of routing variables and link metrics. In this case, communication links with smaller routing metrics are used intensively, and those with larger metrics are used less intensively.

A slightly different approach to minimizing the network links utilization is proposed, for example, in [36,37]. They propose to minimize the upper bound of all network links utilization to avoid the situation where some links will be overloaded and some will be underloaded. Such solutions meet the requirements of the Traffic Engineering (TE) concept, which was designed to ensure a balanced use of network resources such as the bandwidth of communication links and buffer queues on routers in the process of solving traffic management problems (routing, link, and buffer resource allocation). In this case, as shown in [36,37], the optimality criterion may take the form:

(22) $$\underset{x,\alpha }{\min}\;\alpha ,$$

where α is the upper bound for the values of the network link utilization, which must meet the conditions

(23) $$\frac{\sum_{k\in K}\lambda_{k}^{\left\langle req\right\rangle }x_{i,j}^{k}}{\varphi_{i,j}}<\alpha \leq 1,\;{\;w}_{i,j}\in W.$$

In the left part of Inequality (23) is an expression for calculating the utilization (7) of the communication link modeled by the edge $w_{i,j}$.

The application of the described routing model, which is represented by Expressions (1)–(20), allows obtaining in analytical form expressions for the calculation of key QoS indicators (10) and (14). These expressions can serve as a basis for formulating the optimality criteria of the second group. For example, the optimality criterion of network solutions can be a minimum weighted sum of the average end-to-end packet delays:

(24) $$J_{\tau }=\sum_{k\in K}pr^{k}\tau_{MP}^{k}\to \min,$$

where $pr^{k}$ is the priority of the kth packet flow.

The application of Criterion (24) is particularly relevant in the process of using the policy of immediate packet transmission EF PHB (Expedited Forwarding Per-Hop Behavior) that, according to the DSCP (Differentiated Services Code Point) method, determines the highest priority of user requests. The higher the packet flow priority, the lower the average end-to-end delay when they are transmitted in the network [26,27].

When transmitting data traffic that is sensitive to probable packet loss, it is advisable to use a criterion that delivers the maximum of the following object function:

(25) $$J_{p}=\sum_{k\in K}pr^{k}\lambda_{k}^{\left\langle req\right\rangle }b^{k}\to \max.$$

The object function (25) characterizes the total amount of data delivered (not lost) in the network for all flows weighted relative to the priority of packets. The higher the priority of a packet flow, the less loss level they will have under the delivery to the receiving router in the network.

Due to the multiservice nature of modern networks, most packet flows generated by the respective network applications are sensitive to the values of the set of QoS indicators. Therefore, in some cases, it is advisable to use a combination of criteria (24) and (25):

(26) $$J_{\tau p}=\sum_{k\in K}\left[h_{\tau }^{k}pr^{k}\tau_{MP}^{k}-h_{p}^{k}pr^{k}\lambda_{k}^{\left\langle req\right\rangle }b^{k}\right]\to \min,$$

where $h_{\tau }^{k}$ and $h_{p}^{k}$ are the weighting coefficients, which, firstly, determine the degree of sensitivity of the kth flow to the values of the end-to-end average delay and the probability of packet loss. Secondly, weights equalize the dimensionality of the physical quantities contained in Expression (26).

The more sensitive the kth flow of packets to the selected QoS indicator, the higher the value of the corresponding weighting coefficient. For example, information for the formation of coefficients $h_{\tau }^{k}$ and $h_{p}^{k}$ can be taken from the corresponding four bits of the Type of Service (ToS) byte, which is contained in the header of each IP packet (Table 2). This byte contains information about the IP priority or DSCP packet code [26,27].

The use of optimality criteria for routing solutions, which explicitly contain the values of specific QoS indicators, does not prohibit the introduction of the relevant QoS conditions into the structure of the optimization problem: for example, (8), (9), and (20) in the presence of precise requirements (norms) relative to the boundary (minimum or maximum) values of the selected QoS indicators: $\lambda_{k}^{\left\langle req\right\rangle }$, $\tau_{\left\langle TH\right\rangle }^{k}$, and $p_{\left\langle TH\right\rangle }^{k}$.

The main advantage of using the optimality criteria of the first group is their linearity and the associated low computational complexity of obtaining final routing solutions. However, the main disadvantage of this approach is the indirect effect of the obtained solutions on the values of most QoS indicators. On the other hand, in the criteria of optimality of the second group, the advantages and disadvantages are the opposite concerning the first group’s solutions. Therefore, the third group of criteria for the optimal solution of routing problems, which is based on a combination of criteria of the first and second groups, aims to complement the advantages and minimize the disadvantages of these routing solutions by expanding their scope. At the same time, the criteria of the third group can be used in cases when not all QoS indicators can be obtained from analytical expressions for their inclusion in the criteria. Then, the different parts of the objective function, the minimum or maximum of which is the essence of the optimality criterion, may be responsible for either determining a particular QoS indicator or considering, for example, the utilization of network links.

## 6. Evaluation of the Tensor Flow-Based Routing Model and Comparative Analysis of QoS Routing Problem Solutions

To assess the effectiveness of routing solutions in the network, we perform a comparative analysis of the proposed tensor model with known approaches for different network structures and QoS requirements. The following routing models were subject to comparison:

- Model 1 is an improved tensor network routing model represented by Expressions (1), (3)–(8), QoS Conditions (9), (10), and (20) and the optimality criterion (24) aimed at minimization weighted sum of the average end-to-end packet delays;
- Model 2 is a flow-based routing model represented by Expressions (1), (3)–(8), and the optimality criterion (25) aimed at the maximization of the weighted amount of data delivered (lost) in the network relative to the priority of packets;
- Model 3 is a flow-based routing model represented by Expressions (1), (3)–(8), and the quadratic analogue of the optimality criterion (21) aimed at the minimization of the conditional cost of routes with link routing metrics similar to OSPF protocol, i.e., the inverse of the link bandwidth;
- Model 4 is a flow-based routing model represented by Expressions (1), (3)–(8), (23), and the optimality criterion (22), which meets the requirements of the Traffic Engineering concept, namely minimization of the upper bound of network link utilization.

**Figure 1:**
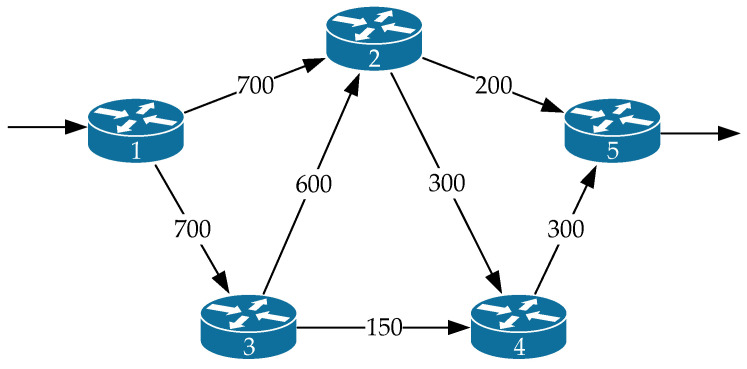
The first variant of the network structure under investigation.

**Figure 2:**
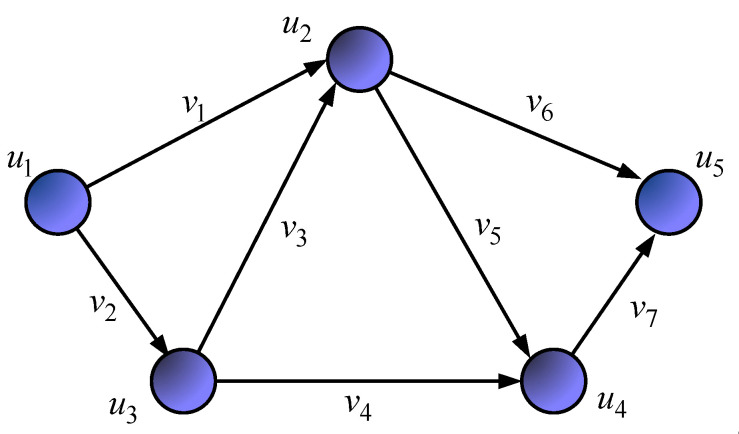
An example of a one-dimensional network S that models the network structure.

Figure 1 shows the *first variant of the network structure* under investigation. The general research technique will be demonstrated by an example in which the following initial data are defined:

- The network structure is presented in Figure 1, which corresponds to the one-dimensional network shown in Figure 2;
- In Figure 1, in the gaps of communication links, their bandwidth (1/s) is indicated;
- The first router was the source node, and the fifth one was the destination node of the packet flow;
- The operation of each of the router interfaces was modeled by the queuing system M/M/1/N.

**Figure 3:**
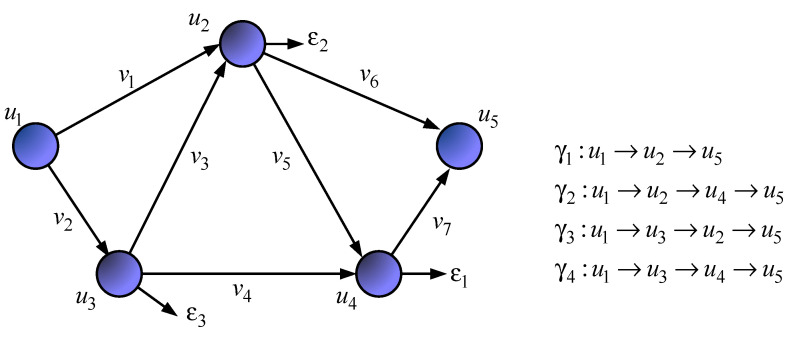
An example of determining the basis of interpolar paths and internal node pairs in the network S.

The network structure (Figure 1) defines a seven-dimensional geometric space. Meanwhile, Figure 2 shows an example of choosing the basis (coordinate system) of edges, and Figure 3 illustrates the basis of independent interpolar paths and internal node pairs.

Then, the matrix of the covariant transformation of coordinates of the introduced tensors will be of the form:

$$A=\|\begin{array}{rrrrrrr} 0 & 0 & 0 & 0 & 0 & 1 & 0\\ 1 & 0 & 0 & -1 & 0 & 0 & 1\\ 0 & 0 & 0 & 0 & 0 & 1 & -1\\ 0 & 1 & 0 & -1 & 1 & 0 & -1\\ 0 & 0 & 0 & 0 & 1 & -1 & 0\\ 0 & 0 & 0 & 1 & 0 & -1 & 0\\ 0 & 0 & 1 & 0 & -1 & 0 & 0 \end{array}\|,$$

where $A={\left[C^{t}\right]}^{-1}$ [17].

In the first case, the influence of the type of optimality criterion on the final nature of QoS routing solutions of one packet flow has been investigated, which was organized using the model (1), (3)–(8), and based on the proposed QoS conditions (9), (10), and (20). The following data were used as input:

$$\lambda^{\left\langle req\right\rangle }=10÷485\;1/s;\;\tau_{\left\langle TH\right\rangle }=80\;\mathrm{ms};\;p_{\left\langle TH\right\rangle }=0.02;\;N=30.$$

**Figure 4:**
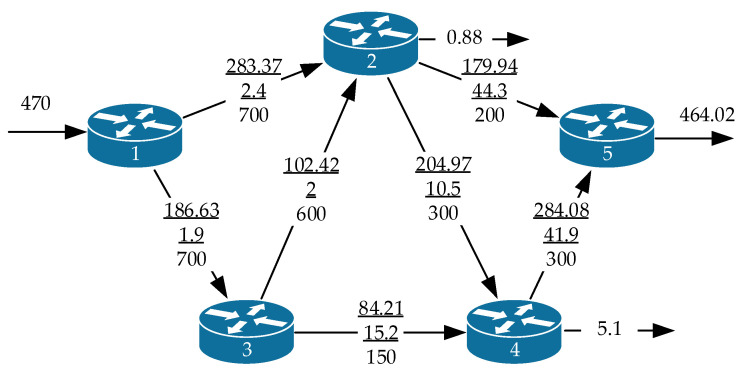
Order of QoS routing of the packet flow under optimality criterion (24).

Under the conditions of using the optimality criterion (24), which minimizes the average end-to-end packet delay in the network, the QoS routing process used all available paths (Figure 4). Additionally, Figure 4 demonstrates in the communication link breaks the following data (top to bottom): packet flow intensity, average packet delay, and bandwidth. In the case of packet loss near the corresponding router output interface, Figure 4 and the following ones show the intensity of the discarded packet flow in the gap of the arrow.

Table 3 presents the characteristics of the calculated routes (Figure 4).

**Figure 5:**
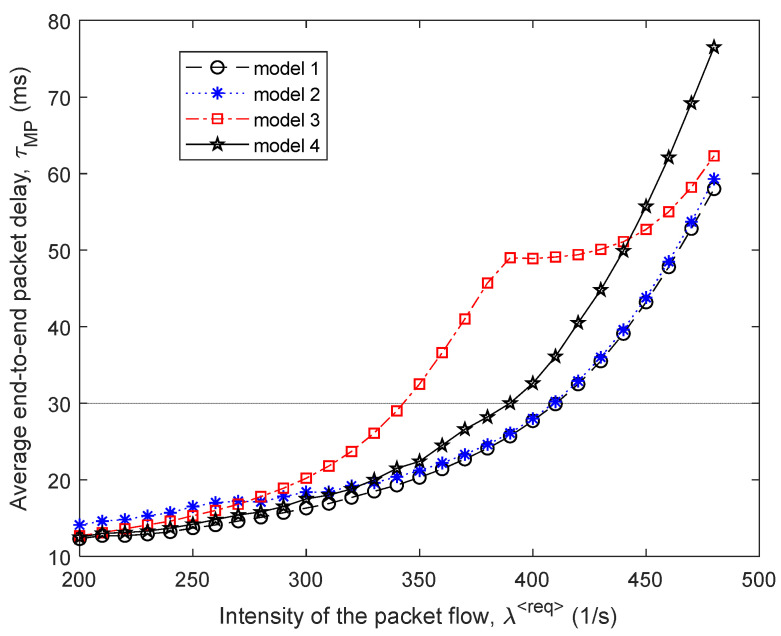
Dependence of the average end-to-end packet delay $\tau_{MP}$ on the intensity of the incoming packet flow $\lambda^{\left\langle req\right\rangle }$ for the compared routing models.

**Figure 6:**
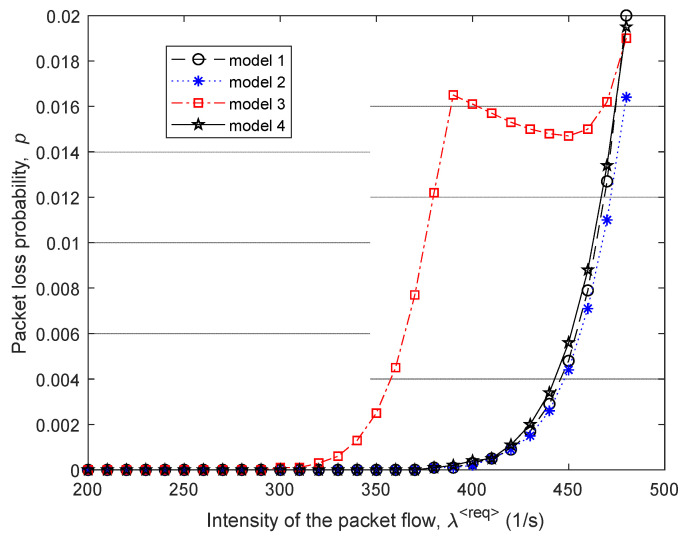
Dependence of the packet loss probability p (9) on the intensity of the incoming packet flow $\lambda^{\left\langle req\right\rangle }$ for the compared routing models.

Then, Figure 5 and Figure 6 show the dependence of the average end-to-end packet delay $\tau_{MP}$ and, accordingly, the probability of packet losses p (9) on the intensity of the incoming packet flow $\lambda^{\left\langle req\right\rangle }$ for the four compared routing models.

**Figure 7:**
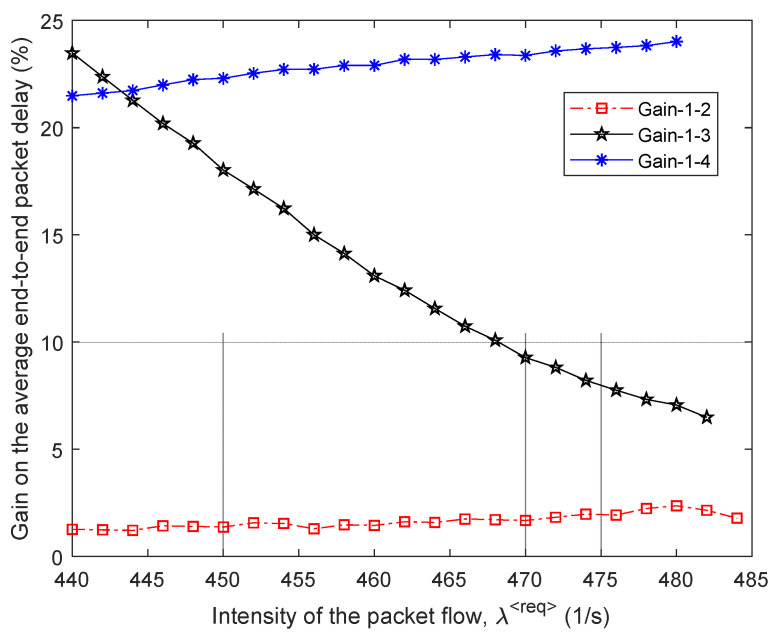
Gain on the average end-to-end packet delay from applying the proposed model (model 1) compared to other routing models depending on the intensity of the incoming packet flow (first network structure).

In Figure 7, the percentage gain in the average end-to-end packet delay ($\tau_{MP}$) from the application of the proposed model 1 compared to other routing models depending on the intensity of the incoming packet flow $\lambda^{\left\langle req\right\rangle }$ is shown. For example, in this figure, the “Gain 1–2” curve indicates the advantages of using model 1 compared to the solution of model 2. Figure 7 also shows the area of high loads when $\lambda^{\left\langle req\right\rangle }=440÷485$ 1/s, because within this range of loads for all models, packet losses have already been observed, but they did not exceed the established tolerances, i.e., p≤0.02 (Figure 6).

Therefore, we can conclude that for the first variant of the network structure (Figure 1), the application of the proposed tensor routing model allows obtaining the following gain for the average end-to-end packet delay around high loads (Figure 7):

- Compared to model 4 on average from 21.5% to 24%;
- Compared to model 3—from 6% to 23.5%;
- Compared to model 2—from 1.5% to 2.5%.

**Figure 8:**
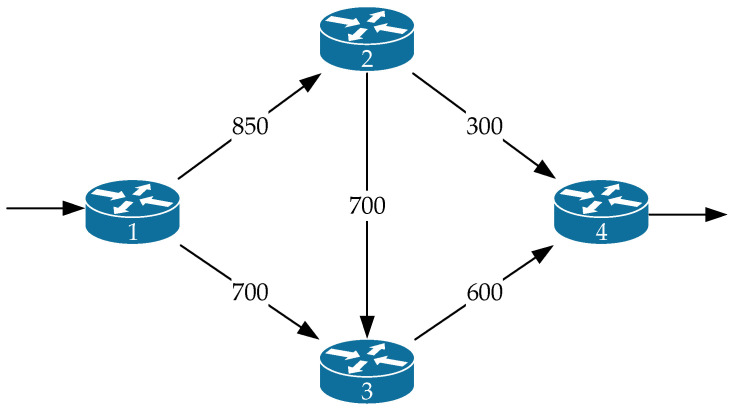
The second variant of the network structure under investigation.

We compared the *second variant of the network structure* we studied to the first network structure (Figure 1). It consists of fewer routers and communication links (Figure 8), where the designation is similar to Figure 1. The source of packets was the first router, and the receiver of packets was the fourth router. The operation of each of the router interfaces was again modeled by the queuing system M/M/1/N under the condition N=25.

The following data were used as input for modeling:

$$\lambda^{\left\langle req\right\rangle }=10÷840\;1/s;\;\tau_{\left\langle TH\right\rangle }=50\;\mathrm{ms};\;p_{\left\langle TH\right\rangle }=0.02;\;N=25.$$

**Figure 9:**
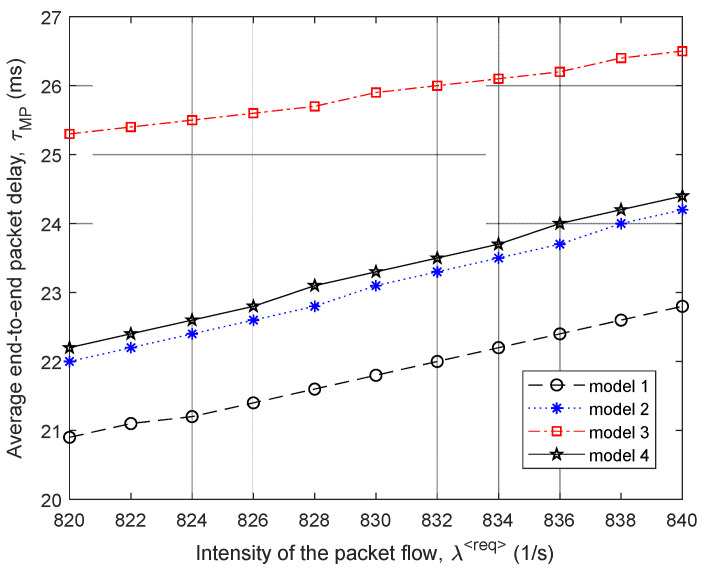
Dependence of the average end-to-end packet delay $\tau_{MP}$ on the intensity of the incoming packet flow $\lambda^{\left\langle req\right\rangle }$ for the compared routing models (second network structure).

**Figure 10:**
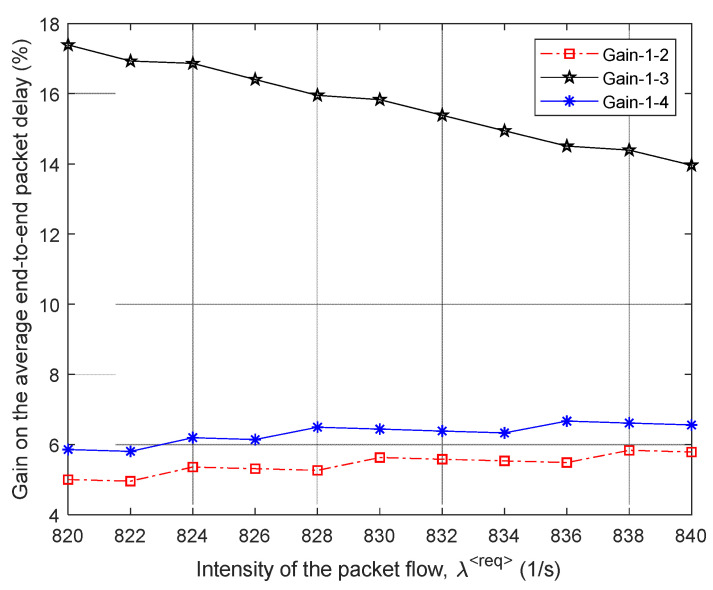
Gain on the average end-to-end packet delay from applying the proposed model (model 1) compared to other routing models depending on the intensity of the incoming packet flow (second network structure).

The same four routing models were studied (Figure 9). Consequently, Figure 9 and Figure 10 show the area of high load on the network when $\lambda^{\left\langle req\right\rangle }=820÷840$ 1/s, because in this case, there were already packet losses, but they did not exceed the established tolerances; i.e., p≤0.02.

As shown by the results of calculations (Figure 9) for the second variant of the network structure (Figure 8), the application of the proposed tensor routing model allows us to obtain the following gain on the average end-to-end packet delay around high loads (Figure 10):

- Compared to model 3 on average from 14% to 17.5%;
- Compared to models 2 and 4—from 5% to 6.5%.

**Figure 11:**
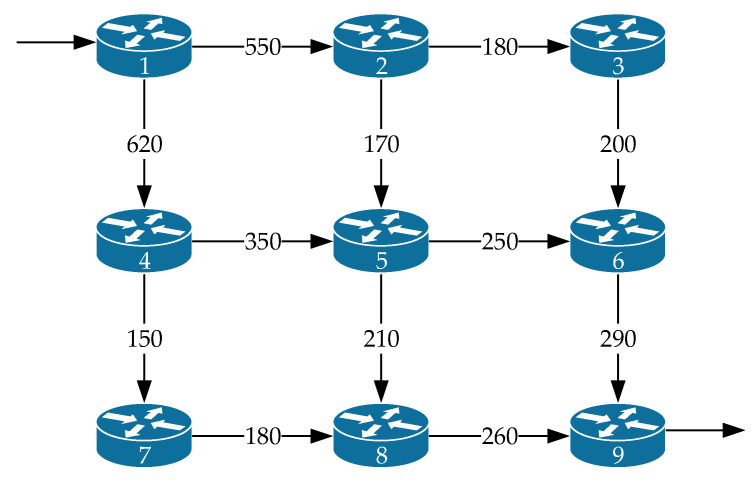
The third variant of the network structure under investigation.

Figure 11 shows the *third variant of the network structure* under investigation. The network contained nine routers and twelve communication links. The notation in Figure 11 is similar to that in Figure 1. The packet flow source node was the first router, and the receiver was the ninth router. The operation of each of the router interfaces was again modeled by the queuing system M/M/1/N, where N=50.

The following data were used as input for modeling:

$$\lambda^{\left\langle req\right\rangle }=10÷485\;1/s;\;\tau_{\left\langle TH\right\rangle }=80\;\mathrm{ms};\;p_{\left\langle TH\right\rangle }=0.03;\;N=50.$$

**Figure 12:**
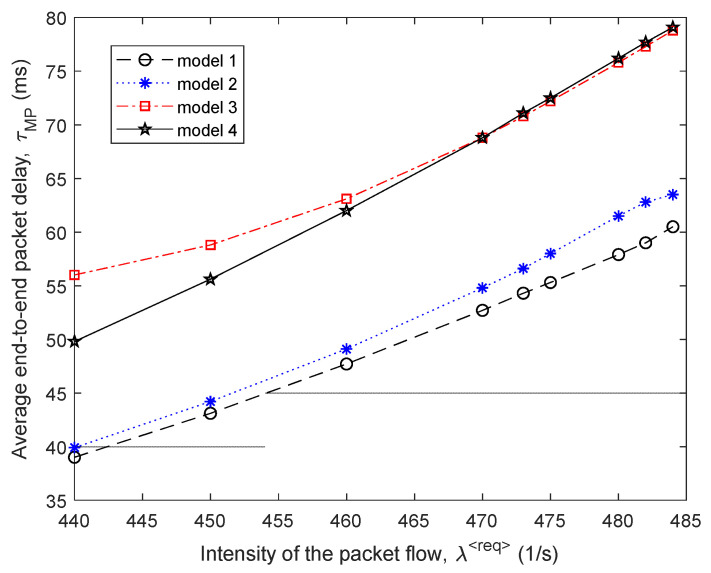
Dependence of the average end-to-end packet delay $\tau_{MP}$ on the intensity of the incoming packet flow $\lambda^{\left\langle req\right\rangle }$ for the compared routing models (third network structure).

The study is still subject to the four routing models mentioned above. In Figure 12, the area of high load on the network when $\lambda^{\left\langle req\right\rangle }=440÷485$ 1/s is presented, because in this case, packet losses have already been observed, but they did not exceed the established tolerances, which for this example are equal to p≤0.03.

**Figure 13:**
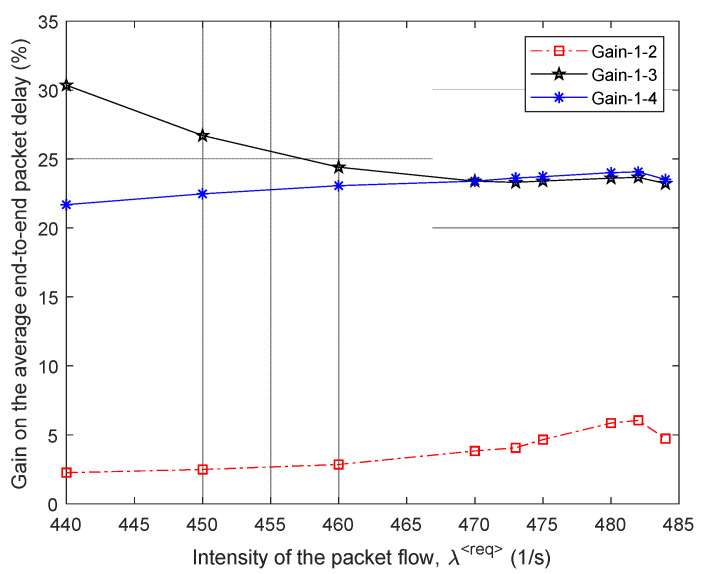
Gain on the average end-to-end packet delay from applying the proposed model (model 1) compared to other routing models depending on the intensity of the incoming packet flow (third network structure).

As shown by the results of calculations (Figure 12) for the third variant of the network structure (Figure 11), the application of the proposed tensor routing model allows us to obtain the following gain on the average end-to-end packet delay around high loads (Figure 13):

- Compared to model 4 on average from 22% to 24%;
- Compared to model 3—from 23% to 30%;
- Compared to model 2—from 3% to 6%.

**Figure 14:**
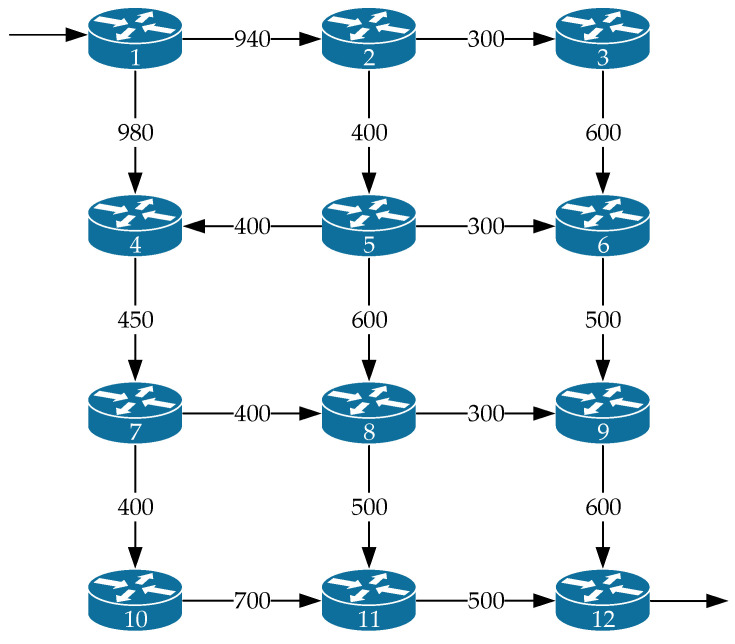
The fourth variant of the network structure under investigation.

Figure 14 shows the *fourth variant of the network structure* under investigation. The network contained twelve routers and seventeen communication links. The notation used in Figure 14 is similar to that in Figure 1. In this example, the source of the packet flow was the first router, the receiver of the packet flow was the twelfth router, and the operation of each of the router interfaces was still modeled by the M/M/1/N queuing system, where N=25.

The following data were used as input for modeling:

$$\lambda^{\left\langle req\right\rangle }=10÷980\;1/s;\;\tau_{\left\langle TH\right\rangle }=100\;\mathrm{ms};\;p_{\left\langle TH\right\rangle }=0.01;\;N=25.$$

**Figure 15:**
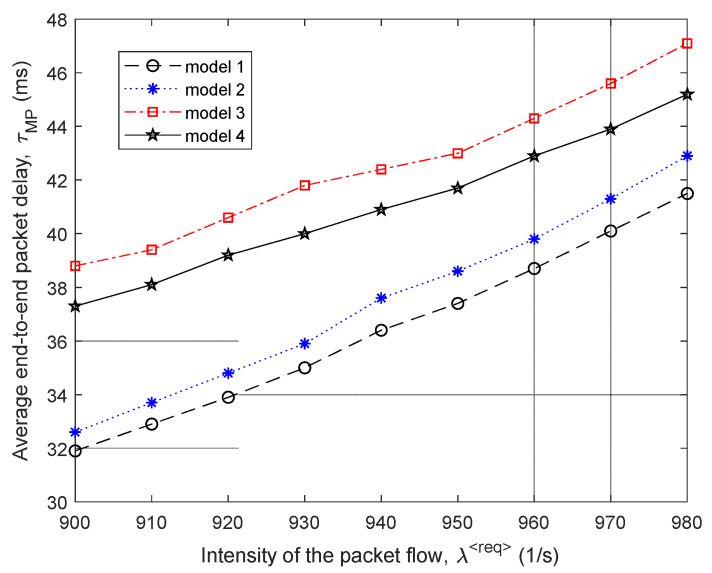
Dependence of the average end-to-end packet delay $\tau_{MP}$ on the intensity of the incoming packet flow $\lambda^{\left\langle req\right\rangle }$ for the compared routing models (fourth network structure).

**Figure 16:**
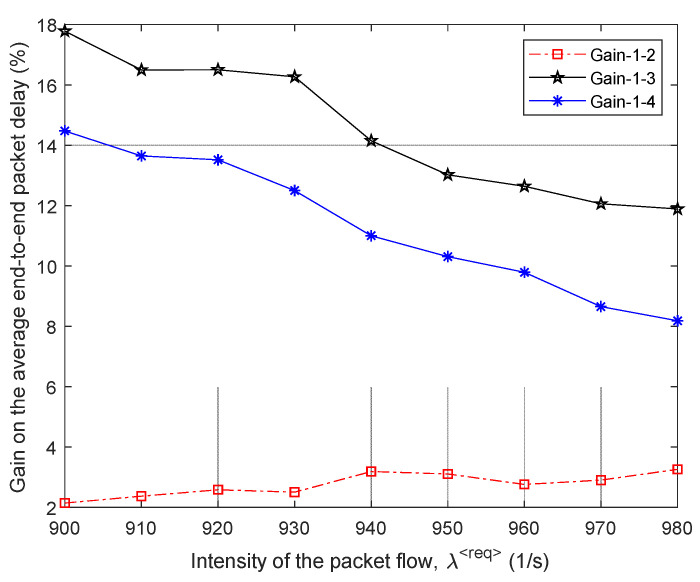
Gain on the average end-to-end packet delay from applying the proposed model (model 1) compared to other routing models depending on the intensity of the incoming packet flow (fourth network structure).

A comparison of the efficiency of the four routing models is shown in Figure 15 and Figure 16 around high load on the network, when $\lambda^{\left\langle req\right\rangle }=900÷980$ 1/s, and the level of packet losses did not exceed the established tolerances, i.e., p≤0.01.

As shown by the results of calculations (Figure 15) for the fourth variant of the network structure (Figure 14), the application of the proposed tensor routing model allows us to obtain the following gain on the average end-to-end packet delay around high loads (Figure 16):

- Compared to model 4 on average from 8% to 15%;
- Compared to model 3—from 12% to 18%;
- Compared to model 2—from 2% to 3%.

Each of the models 1–4 has its mechanism for reducing the average end-to-end packet delay in the network. The proposed model (model 1) works most efficiently; i.e., it provides optimal path calculation and load balancing through optimality criterion, which is directly related to minimizing the priority-weighted end-to-end packet delays of different flows. Model 2 focuses on the indirect improvement of packet delay by organizing such load balancing to maximize network performance. Models 3 and 4, through the used optimality criteria, minimize the utilization of communication links, which also indirectly improves the Quality of Service level, including the average end-to-end packet delay. In model 3, as the optimality criterion, the additive quadratic form of link utilization coefficients is used. However, model 4, which minimizes the upper bound of network link utilization, provides a better order of load balancing according to the average end-to-end packet delay QoS indicator than model 3.

## 7. Conclusions

The article improves the tensor model of the telecommunication network, which is presented in the coordinate system of interpolar paths and internal node pairs. The novelty of the proposed solution is obtaining conditions for Quality of Service in terms of network performance: bandwidth, average end-to-end delay, and the packet loss probability shown in (9), (10), and (20), which are valid for different network load modes, and not only for the mode close to overload. Moreover, the presented model does not require the use of all available communication links and network routes. Thus, applying an advanced network tensor model to solve routing problems can improve the QoS by reducing the average end-to-end packet delay. Furthermore, if necessary, it can ensure the adaptive use of network links and paths to implement both single path and multipath routing.

The formulation of the required QoS conditions in terms of network performance in (8)–(10), and (20) is based on obtaining analytical expressions to calculate the average end-to-end packet delay in the IoT core network (14) and the packet loss probability. In addition, it adequately considers network topology, traffic characteristics, and packet service disciplines at network nodes, particularly in network interface overload conditions. Note that obtaining such expressions also is an essential step in formulating the conditions of ensuring the Quality of Experience (QoE) because the indicators of multimedia quality are a function of the listed indicators of network performance, which is a direction of future research.

In addition, the article proposes a system of optimality criteria of routing solutions in (21) and (24)–(26), which can be used to calculate the optimal values of routing variables. Depending on the specifics of the routing problem in the IoT core network, these criteria focus on the optimal use of available network resources and/or on the differentiated improvement of QoS indicators of network performance to the numerical values of which a particular packet flow is sensitive.

The analysis of the influence of optimality criterion type on the character of QoS routing problem solutions using the offered tensor model of a telecommunication network is carried out. On several computational examples, the improved tensor model of the network both at the level of the proposed QoS conditions (9), (10), and (20), and the considered QoS optimality criteria (24) and (25), confirmed its adequacy in solving problems of multipath routing. The improved network tensor model provided an adaptive response to changes in network load and the type of criterion for optimal routing solutions in terms of providing a set of QoS indicators: transmission rate ($\lambda^{\left\langle req\right\rangle }$), average end-to-end delay ($\tau_{\left\langle TH\right\rangle }$), and packet loss probability ($p_{\left\langle TH\right\rangle }$).

We performed the study and comparative analysis of the proposed tensor model of the network with other known models for solving routing problems. Depending on the chosen network topology, the application of the proposed tensor routing model reduces the average end-to-end packet delay around high loads (Figure 7, Figure 10, Figure 13 and Figure 16). For example, compared to mathematical models based on routing metrics, the gain ranged from 6–12% to 18–30%, while Traffic Engineering solutions gain varied from 5–8% to 21.5–24%.

Our future work will focus on enhancing the tensor flow-based QoS routing models toward QoE support, as well as taking into account the dynamics of changing the network state when conditions of assurance QoS over the set of NP indicators will be functions of time. Moreover, the presented solution can be adapted to solving the technical task of fast rerouting in the case of failures of network elements (links, nodes, and paths). Here, the demanded level of QoS must be provided over the primary and backup routes via implementation of the corresponding schemes of QoS protection (reservation).

## Figures and Tables

**Table 1:** Comparison of existing solutions on QoS routing in IoT, WSN, and SDN-based networks.

Ref.	Contribution	NP Parameter	Field of Application
[28]	The QoS-aware flexible mobility management scheme that classifies flows into four classes has been proposed. Type of solution: heuristic. Advantages: flexible network resource utilization, differential handover for different flow classes, absence of service degradation.	Delay, loss	SDN-based mobile networks
[29]	The multi-constrained QoS resource allocation model based on network calculus and the multi-constrained centralized QoS routing algorithm in SDN has been presented. Type of solution: optimization. Advantages: accurate QoS guarantee, fast routing, better algorithm performance in terms of effective bandwidth utilization rate, path load, end-to-end delay.	Delay, bandwidth	SDN-based streaming media networks
[30]	The Blockchain-Enabled QoS-Based Inter-Autonomous System Routing in SDN, namely RoutingChain, has been proposed. Type of solution: blockchain-based heuristic. Advantages: QoS-based inter-AS coordination eliminating centralized mediators, using blockchain technology in networking, reducing overhead, mitigating privacy/security in inter-AS routing.	Delay, bandwidth	Multi-area SDN networks
[31]	The Cross-layer Optimized Opportunistic Routing (COOR) scheme is proposed to improve communication link reliability and reduce delay for loss-and-delay sensitive WSNs. Two optimization strategies are used: COOR (R) under higher reliability and COOR(P) under longer distance. Type of solution: optimization. Advantages: reducing delay, improving reliability, balancing energy consumption due to full use of the remaining energy in networks aimed at increasing the transmission power of nodes.	Delay, loss	Wireless sensor networks (WSNs)
[32]	A Differentiated Data Aggregation Routing (DDAR) scheme is proposed to reduce energy consumption and guarantee delay under corresponding QoS requirement constraints. Type of solution: optimization. Advantages: reducing delay, improving lifetime, increasing energy efficiency without performance degradation of data transmission.	Delay	Delay sensitive wireless sensor networks
[33]	The Delay-Intolerant Energy-Efficient Routing (DIEER) with sink mobility in Underwater Wireless Sensor Networks has been developed. The DIEER uses a framework for the joint optimization of sink mobility, hold and forward mechanisms, adoptive depth threshold, and data aggregation with pattern matching for reducing nodal propagation delay, maximizing throughput, improving network lifetime, and minimizing energy consumption. Type of solution: optimization. Advantages: reducing the number of re-transmissions, good energy conservation, enhancing throughput.	Delay, bandwidth	Underwater Wireless Sensor Networks (UWSNs)
[34]	A smart collaborative routing protocol with low delay and high reliability is proposed together with forwarding, maintenance, and efficiency strategies created to construct the basic protocol functionalities. Type of solution: heuristic. Advantages: improving the kernel tree routing protocol based on deep learning, reducing the number of hops, using the stabilizing algorithm considering tree topology to save protocol overhead, improving link utilization.	Delay	Industrial IoT (IIoT)
[35]	The SDN-Based Application-aware Distributed adaptive Flow Iterative Reconfiguring (SADFIR) routing protocol has been proposed. Type of solution: optimization. Advantages: load balancing, application-aware data transmission, heterogeneity aware.	Bandwidth	SDN-based IoT

**Table 2:** Variants of the service type field values in the IP packet header.

Value of the ToS Field	QoS Level Requirements
1000	minimum delay
0100	maximum bandwidth
0010	maximum reliability
0001	minimum cost
0000	services without QoS requirements

**Table 3:** Characteristics of the calculated routes for the network structure shown in Figure 4.

$\lambda^{\left\langle req\right\rangle },\;1/s$	$\lambda^{*},\;1/s$	|P|	Characteristics of the Calculated Routes			$\tau_{MP},\;\mathrm{ms}$	***p***
			Route	$\lambda_{p},\;1/s$	$\tau_{p},\;\mathrm{ms}$		
470	464.02	4	$R_{1}\to R_{2}\to R_{5}$	179.94	46.7	52.8	0.0127
			$R_{1}\to R_{2}\to R_{4}\to R_{5}$	100.74	54.8		
			$R_{1}\to R_{3}\to R_{2}\to R_{4}\to R_{5}$	100.61	56.4		
			$R_{1}\to R_{3}\to R_{4}\to R_{5}$	82.73	59.1		

## Data Availability

Not applicable.

## References

[1] White R., Banks E. (2017). Computer Networking Problems and Solutions: An Innovative Approach to Building Resilient, Modern Networks.

[2] Barona López L.I., Valdivieso Caraguay Á.L., Sotelo Monge M.A., García Villalba L.J. (2017). Key Technologies in the Context of Future Networks: Operational and Management Requirements. Future Internet.

[3] Li Y., Su X., Ding A.Y., Lindgren A., Liu X., Prehofer C., Riekki J., Rahmani R., Tarkoma S., Hui P. (2020). Enhancing the Internet of Things with Knowledge-Driven Software-Defined Networking Technology: Future Perspectives. Sensors.

[4] Kron G. (1965). Tensor Analysis of Networks.

[5] Kron G. (1963). Diakoptics; the Piecewise Solution of Large-Scale System.

[6] Orús R. (2014). A practical introduction to tensor networks: Matrix product states and projected entangled pair states. Ann. Phys..

[7] Phan A.-H., Cichocki A., Uschmajew A., Tichavský P., Luta G., Mandic D.P. (2020). Tensor Networks for Latent Variable Analysis: Novel Algorithms for Tensor Train Approximation. IEEE Trans. Neural Netw. Learn. Syst..

[8] Jin D., Wu Y., Yan G., Wang Y., Ma Q., Li J. A Community Detecting Algorithm Based on Modular Tensor in Temporal Network. Proceedings of the 2018 IEEE 16th International Conference on Dependable, Autonomic and Secure Computing, 16th International Conference on Pervasive Intelligence and Computing, 4th International Conference on Big Data Intelligence and Computing and Cyber Science and Technology Congress(DASC/PiCom/DataCom/CyberSciTech).

[9] Chen Y.W., Guo K., Pan Y. Robust supervised learning based on tensor network method. Proceedings of the 2018 33rd Youth Academic Annual Conference of Chinese Association of Automation (YAC).

[10] Xie K., Wang L., Wang X., Xie G., Wen J., Zhang G., Cao J., Zhang D. (2018). Accurate Recovery of Internet Traffic Data: A Sequential Tensor Completion Approach. IEEE/ACM Trans. Netw..

[11] Tsai K.C., Zhuang Z., Lent R., Wang J., Qi Q., Wang L.C., Han Z. (2021). Tensor-Based Reinforcement Learning for Network Routing. IEEE J. Sel. Top. Signal Process..

[12] Wang X., Yang L.T., Kuang L., Liu X., Zhang Q., Deen M.J. (2019). A Tensor-Based Big-Data-Driven Routing Recommendation Approach for Heterogeneous Networks. IEEE Netw..

[13] Sun K., Yuan L., Xu H., Wen X. (2020). Deep Tensor Capsule Network. IEEE Access.

[14] Strelkovskaya I.V., Solovskaya I.N. (2013). Tensor model of multiservice network with different classes of traffic service. Radioelectron. Commun. Syst..

[15] Strelkovskaya I., Solovskaya I. Tensor decomposition in the structure optimization tasks of LTE/MVNO networks. Proceedings of the 2014 IEEE International Black Sea Conference on Communications and Networking (BlackSeaCom).

[16] Lemeshko O., Kovalenko T., Nevzorova O., Ilyashenko A., Radivilova T., Ageyev D., Kryvinska N. (2021). Diakoptical Method of Inter-area Routing with Load Balancing in a Telecommunication Network. Data-Centric Business and Applications. Lecture Notes on Data Engineering and Communications Technologies.

[17] Lemeshko A.V., Evseeva O.Y., Garkusha S.V. A tensor model of multipath routing based on multiple QoS metrics. Proceedings of the 2013 International Siberian Conference on Control and Communications (SIBCON).

[18] Lemeshko O., Yevdokymenko M., Naors Y. (2018). Development of the tensor model of multipath QoE-routing in an infocommunication network with providing the required quality rating. East. Eur. J. Enterp. Technol..

[19] Lemeshko O., Yevdokymenko M., Yeremenko O., Mersni A., Segeč P., Papán J. Quality of Service Protection Scheme under Fast ReRoute and Traffic Policing Based on Tensor Model of Multiservice Network. Proceedings of the 2019 International Conference on Information and Digital Technologies (IDT).

[20] Lemeshko O., Yeremenko O., Yevdokymenko M., Hailan A.M., Hu Z., Petoukhov S., Dychka I., He M. (2020). Tensor Multiflow Routing Model to Ensure the Guaranteed Quality of Service Based on Load Balancing in Network. Proceedings of the International Conference on Computer Science, Engineering and Education Applications.

[21] Yeremenko O. (2016). Development of the dynamic tensor model for traffic management in a telecommunication network with the support of different classes of service. Eur. J. Enterp. Technol..

[22] Lemeshko O.V., Yeremenko O.S., Hailan A.M. QoS solution of traffic management based on the dynamic tensor model in the coordinate system of interpolar paths and internal node pairs. Proceedings of the 2016 International Conference Radio Electronics & Info Communications (UkrMiCo).

[23] Lemeshko O., Yevdokymenko M. (2020). Advanced tensor approach to fast reroute with quality of service protection under multiple parameters. Inf. Telecommun. Sci..

[24] Greengard S. (2021). The Internet of Things, revised and updated edition.

[25] Hanes D., Salgueiro G., Grossetete P., Barton R., Henry J. (2017). IoT Fundamentals: Networking Technologies, Protocols, and Use Cases for the Internet Of Things.

[26] Barreiros M., Lundqvist P. (2016). QOS-Enabled Networks: Tools and Foundations.

[27] Goralski W. (2017). The Illustrated Network: How TCP/IP Works in a Modern Network.

[28] Kyung Y., Kim T.-K. (2020). QoS-Aware Flexible Handover Management in Software-Defined Mobile Networks. Appl. Sci..

[29] Zhu S., Sun Z., Lu Y., Zhang L., Wei Y., Min G. (2019). Centralized QoS Routing Using Network Calculus for SDN-Based Streaming Media Networks. IEEE Access.

[30] Karakus M., Guler E. RoutingChain: A Proof-of-Concept Model for a Blockchain-Enabled QoS-Based Inter-AS Routing in SDN. Proceedings of the 2020 IEEE International Black Sea Conference on Communications and Networking (BlackSeaCom).

[31] Xu X., Yuan M., Liu X., Liu A., Xiong N.N., Cai Z., Wang T. (2018). A Cross-Layer Optimized Opportunistic Routing Scheme for Loss-and-Delay Sensitive WSNs. Sensors.

[32] Li X., Liu W., Xie M., Liu A., Zhao M., Xiong N.N., Zhao M., Dai W. (2018). Differentiated Data Aggregation Routing Scheme for Energy Conserving and Delay Sensitive Wireless Sensor Networks. Sensors.

[33] Latif K., Javaid N., Ullah I., Kaleem Z., Abbas Malik Z., Nguyen L.D. (2020). DIEER: Delay-Intolerant Energy-Efficient Routing with Sink Mobility in Underwater Wireless Sensor Networks. Sensors.

[34] Zhu M., Chang L., Wang N., You I. (2020). A Smart Collaborative Routing Protocol for Delay Sensitive Applications in Industrial IoT. IEEE Access.

[35] Shafique A., Cao G., Aslam M., Asad M., Ye D. (2020). Application-Aware SDN-Based Iterative Reconfigurable Routing Protocol for Internet of Things (IoT). Sensors.

[36] Wang N., Ho K.H., Pavlou G., Howarth M. (2008). An overview of routing optimization for internet traffic engineering. IEEE Commun. Surv. Tutor..

[37] Lee Y., Seok Y., Choi Y., Kim C. A constrained multipath traffic engineering scheme for MPLS networks. Proceedings of the 2002 IEEE International Conference on Communications; ICC 2002 (Cat. No.02CH37333).

[38] Lemeshko O., Yeremenko O. Dynamic presentation of tensor model for multipath QoS-routing. Proceedings of the 2016 13th International Conference on Modern Problems of Radio Engineering, Telecommunications and Computer Science (TCSET).

[39] Yeremenko O., Lemeshko O., Tariki N., Hailan A.M. Research of optimization model of fault-tolerant routing with bilinear path protection criterion. Proceedings of the 2017 2nd International Conference on Advanced Information and Communication Technologies (AICT).

[40] Lemeshko O., Yevdokymenko M., Yeremenko O., Shapovalova A., Hu Z., Petoukhov S., Dychka I., He M. (2020). Investigation of Load-Balancing Fast ReRouting Model with Providing Fair Priority-Based Traffic Policing. Proceedings of the International Conference on Computer Science, Engineering and Education Applications.

